# The effects of pharmacomechanical thrombectomy on novel complete blood count parameters in deep vein thrombosis: A retrospective study

**DOI:** 10.1097/MD.0000000000033008

**Published:** 2023-02-17

**Authors:** Ali Ümit Yener, Adnan Yalçinkaya, Özlem Yener, Ekin Can Çelik, Onur Hanedan, Mustafa Cüneyt Çiçek, Ömer Faruk Çiçek

**Affiliations:** a Department of Cardiovascular Surgery, University of Health Science Antalya Education and Training Hospital, Antalya, Turkey; b Department of Radiology, Atatürk State Hospital, Antalya, Turkey; c Department of Cardiovascular Surgery, University of Health Science AhiEvran Education and Training Hospital, Trabzon, Turkey; d Department of Cardiovascular Surgery, Konya City Hospital, Konya, Turkey; e Department of Cardiovascular Surgery, Selçuk University Faculty of Medicine, Konya, Turkey.

**Keywords:** catheter-based interventions, deep vein thrombosis, mechanical thrombectomy, neutrophil-lymphocyte ratio, platelet-lymphocyte ratio

## Abstract

This study aimed to investigate the effects of pharmacomechanical thrombectomy on novel complete blood count parameters in deep venous thrombosis. This retrospective study included 242 patients aged >18 years who were treated for deep venous thrombosis. Patients were grouped as follows: group 1 was accepted as having interventional operations (n = 123) and group 2 was accepted as having only medical advice (n = 119). Routine complete blood count parameters, the neutrophil-to-lymphocyte ratio (NLR), and the platelet-to-lymphocyte ratio (PLR) were compared. There was no difference between the groups in terms of admission hemoglobin, hematocrit, mean platelet volume, NLR and PLR (*P* = .11, *P* = .24, *P* = .55, *P* = .93, and *P* = .96, respectively). In the pharmacomechanic thrombectomy group, NLR and PLR were significantly reduced after intervention when compared to the admission values (*P* < .001 and *P* < .001, respectively). However, the NLR and PLR values of medically treated patients did not differ significantly from their baseline values (*P* = .16 and *P* = .08, respectively). In this study, we effectively removed the thrombus load in blocked proximal veins using pharmacomechanical thrombectomy and observed a significant decrease in NLR and PLR, which are current, inexpensive, and accessible parameters.

## 1. Introduction

Deep vein thrombosis (DVT) is the blockage of the deep vessels by blood clots. It affects all races and it is observed in 1 person out of 1000 people with more than 250,000 new cases reported annually.^[1]^ Three effective parameters contributing to the development of the disease were described by Virchow in 1863. These effects included endothelial damage, decreased blood viscosity (stasis), and hypercoagulability.^[2]^

DVT is a preventable cause of death. Although it usually occurs in patients > 40 years of age, it can also be observed in young patients. The risk factors include major surgical operations, immobilization, genetic diseases that may cause coagulation tendencies, hormonal drugs, and pregnancy. The 2 most important complications of DVT are pulmonary embolism and post thrombotic syndrome.^[3]^ In order to avoid these 2 undesirable conditions, it is necessary to diagnose the disease and start treatment as soon as possible. The conventional treatment for DVT involves the use of oral anticoagulants. The use of the method of decomposition of thrombosis through the catheter, which was first described in 1997, has increased in recent years in the early treatment of DVT.^[4]^ Pharmacomechanical thrombectomy is a useful and effective technique.^[5]^ With this technique, the thrombus load on the venous system is reduced, the venous thrombus is generally cleaned, venous drainage is provided and unwanted complications are avoided.^[6]^

In recent years, the ratio of neutrophils and platelets to lymphocytes (the ratio of N/L and P/L) has been considered an indicator of systemic inflammation and has been shown to be associated with the prognosis of many cardiovascular diseases, malignancies, and chronic inflammatory diseases.^[7]^ It is stated that the neutrophil-lymphocyte ratio increases in cardiovascular diseases and pulmonary embolism cases.^[8,9]^ A higher platelet-lymphocyte ratio in cancer patients and patients undergoing head-neck surgery increases the probability of venous thromboembolism (VTE).^[10,11]^ A higher ratio of neutrophil-lymphocyte and platelet-lymphocyte together increases the risk of developing VTE in patients who have undergone surgery.^[12]^ A recent study by Peng et al showed that in patients who have undergone oral cancer surgery, a high neutrophil-to-lymphocyte ratio (NLR) level, which is suggestive of inadequate immunity and nutrition, increases the risk of DVT.^[13]^

The mean platelet volume (MPV) is considered an indicator of platelet function and activation, and higher values of MPV have been shown to be independent risk factors for acute myocardial infarction, renal artery stenosis, diabetes mellitus, hypertension, and hyperlipidemia.^[14]^

In this study, we investigated the effects of 2 different treatment approaches in DVT (Pharmacomechanical thrombectomy and medical follow-up) on neutrophil-lymphocyte ratio, platelet-lymphocyte ratio, and mean platelet volume.

## 2. Methods

First, it was a retrospective study. Two hundred forty-two patients, who are over 18 years old and treated for deep venous thrombosis between January 2017 and January 2020, were included in the study. The hospital database was scanned and the acceptance criteria for the patients were based on the Declaration of Helsinki. The procedures were performed by a single surgeon at a single center, and the study was designed as a retrospective study. Patients data was provided from the hospital’s anonymous database by the data protection officer ensuring the privacy of the patients identity. Patients who were referred to a cardiovascular surgery outpatient clinic and diagnosed with DVT using Doppler ultrasonography were included in this study. Patients with chronic DVT, who had complaints for more than 2 weeks, had new findings of chronic DVT, superficial and/or thrombosis at the knee level, and were under the age of 18 years were excluded from the study. Written informed consent was obtained from all patients according to the treatment protocol. This study was conducted in accordance with the STROBE statement.

Doppler ultrasound of the patients was evaluated in detail. Both superficial and deep venous systems of the lower extremities, iliac veins, and inferior vena cava (IVC) were imaged and the findings were recorded. Patients were classified according to the location of the thrombosis, as in the femoral and iliac veins. Informed consent was obtained from patients who had iliofemoral DVT and were admitted to the hospital for interventional procedures (Group 1, n = 123). Patients who had iliofemoral DVT and did not undergo the interventional procedure, and those who had femoral DVT and received medical treatment were included (Group 2, n = 119).

Demographic findings, comorbidities, and complete blood counts (CBC) of the patients were recorded. Hemoglobin, Hematocrit, MPV, NLR, and platelet-to-lymphocyte ratio (PLR) were recorded during the patient’s first hospitalization.

Pharmacomechanical thrombectomy was performed in 123 patients. Two patients had bilateral iliofemoral DVT. The VCI filter was introduced through the jugular vein. During the same session, the interventional procedures were performed on both legs in the prone position. The remaining patients underwent interventions for the affected extremities.

### 2.1. Statistical analysis

Data were analyzed using the statistical package for social sciences for Windows 21.0 software (IBM SPSS Inc., Chicago, IL). Normal distribution of the data was evaluated using the Kolmogorov-Smirnov test. Numerical variables with normal distributions are shown as minimum-maximum values. Categorical variables are presented as numbers and percentages. Differences in normally distributed variables between groups were evaluated using the student *t* test. Categorical variables between the groups were evaluated using the chi-squared test. Comparisons within groups were performed using a paired sample test. Statistical significance was set at *P* ≤ .05.

### 2.2. Interventional technique

Group 1 patients underwent pharmacomechanical thrombectomy. First, patients were placed in the supine position. Under fluoroscopy, the temporary vena cava filter Inca (Invamed, Ankara, Turkey) was introduced through the femoral vein and positioned in the IVC. Subsequently, the patient was placed in the prone position and an introducer sheath was introduced into the popliteal vein of the affected leg using Doppler USG as a guidance modality. Heparin (5000 IU) was administered. We performed venography to assess the venous lumen and marked any obstructed segments as we waited to achieve adequate anticoagulation. After, injecting a 1/10th diluted 1 mg tissue plasminogen activator (TPA) with serum physiologic (20 cm^3^) and waiting for 3 minutes, mechanical thrombectomy device Mantis (Invamed, Ankara, Turkey) was introduced along the vein under imaging guidance and thrombus aspiration device Dovi (Invamed, Ankara, Turkey) was connected to the aspiration terminal of the sheath. Mechanical thrombectomy, aspiration, and TPA infusion (20 cm^3^/1 mg, totally 200 cm^3^) were performed simultaneously until obstructive thrombi were cleared. Then Mantis and Dovi removed from the introducer sheath. Finally, the pharmacomechanical thrombectomy catheter Viper (Invamed, Ankara, Turkey) catheter was positioned with its tip at the most proximal point of the iliac vein. The patient transferred to the intensive care unit and TPA infused 1 mg/h for 24 hours. After 24 hours, Viper was removed along with the introducer sheath. Under fluoroscopy the temporary vena cava filter Inca was removed and the patient was transferred to the recovery room.

Patients in group 2 had iliofemoral or femoral DVT. Patients with iliofemoral DVT were not treated with pharmacokinetic thrombectomy according to their choice, while patients with femoral DVT were not included among the procedure indications. These patients were treated with medications. All the patients in Group 1 and Group 2 received venoprotectiveagent (Diosmin + Hesperidin 500 mg 2 × 1) and anticoagulation agent (0.1 mg/kg enoxaparin sodium 2 × 1, 10 days).

All patients were evaluated at the outpatient clinic on the 10^th^ days of initial treatment for pharmacomechanical thrombectomy or medical treatment. Physical examinations and CBC tests were performed. Warfarin was prescribed to all patients after the first follow-up for 6 months.

## 3. Results

**Figure 1.**
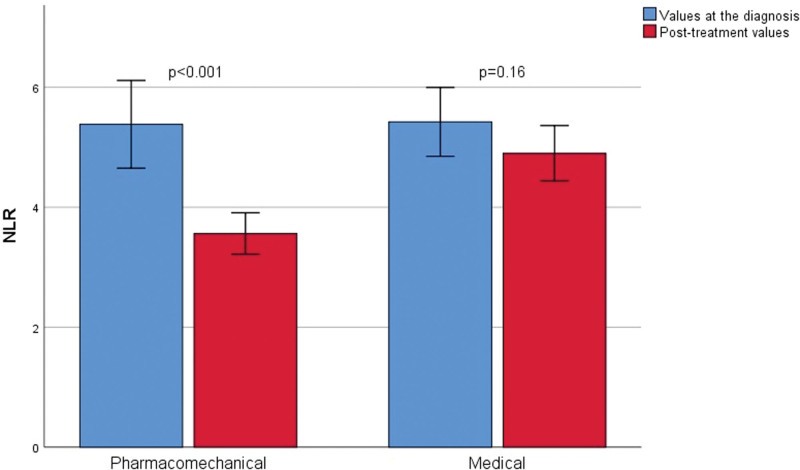
Comparison of admission and control NLR in both pharmacomechanical thrombectomy and medically treated patient groups separately (with 95% CI error bars). NLR = neutrophil-to-lymphocyte ratio.

**Figure 2.**
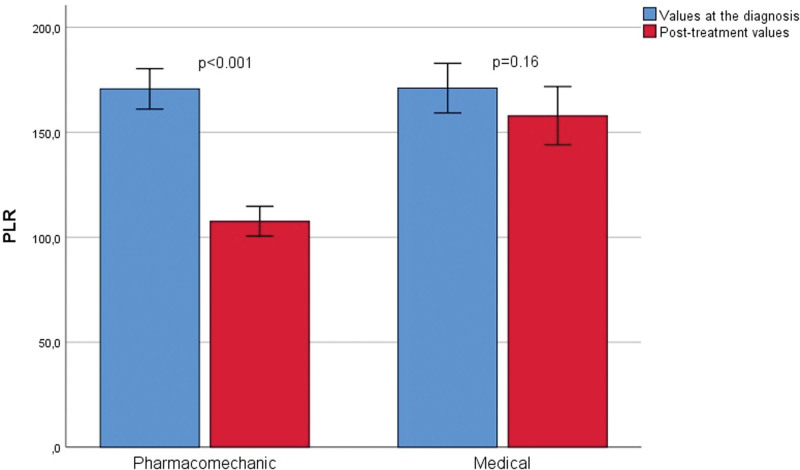
Comparison of admission and control PLR in both pharmacomechanical thrombectomy and medically treated patient groups separately (with 95% CI error bars). PLR = platelet-to-lymphocyte ratio.

A total of 242 patients were included in this study. A total of 123 patients under went pharmacomechanical thrombectomy and 119 received medical therapy. The data were retrospectively evaluated. The pharmacomechanical thrombectomy group (group 1) and medically treated patient group (group 2) were compared in terms of various parameters. There was no difference between the groups in terms of age, sex distribution, diabetes mellitus, hypertension, malignancy, DVT direction, hemoglobin, hematocrit, MPV, NLR, and PLR values (Table 1). In terms of DVT localization, although iliofemoral DVT was present in all patients who underwent pharmacomechanical thrombectomy, iliofemoral DVT was observed in 64 (53.8%) patients and femoral DVT was observed in 55 (46.2%) patients who were medically treated. A significant difference was observed between groups (*P* < .001). There was no difference between the groups in terms of admission hemoglobin, hematocrit, MPV, NLR, and PLR. Table 2 shows the comparison of the posttreatment hemoglobin, hematocrit, MPV, NLR, and PLR values between pharmacomechanical thrombectomy and medically observed patient groups. There was no difference between the groups in terms of follow-up hemoglobin, hematocrit, and MPV (*P* = .11, *P* = .2, and *P* = .06, respectively). However, the posttreatment NLR and PLR values were significantly lower in the pharmacomechanic thrombectomy group when compared with medically treated patients (*P* < .001 and *P* < .001, respectively). The admission and follow-up values of laboratory tests were statistically compared as dependent variables within the groups (Table 3). MPV values were reduced at follow-up compared to admission values in both the pharmacomechanical thrombectomy and medically treated groups; however, this difference was not statistically significant. On the other hand, NLR and PLR values were also found to be decreased in the follow-up rather than at admission in the medically treated patients group, but this difference was not found to be statistically significant (*P* = .16 and *P* = .08, respectively) however, the posttreatment NLR and PLR values were significantly decreased when compared with the admission values in the pharmacomechanic thrombectomy group (*P* < .001 and *P* < .001, respectively) (Figs. 1 and 2).

**Table 1:** Demographic characteristics and CBC parameters of the study group.

	Total (n = 242)	Pharmacomechanic (n = 123)	Medical (n = 119)	*P* value
Age	53.61 ± 16.82	52.03 ± 16.73	55.24 ± 16.84	.14
Sex				.52
Male	119 (49.2%)	58 (47.2%)	61 (51.3%)	
Female	123 (50.8%)	65 (52.8%)	58 (48.7%)	
Diabetes mellitus	27 (11.2%)	10 (8.1%)	17 (14.3%)	.19
Hypertension	76 (31.4%)	40 (32.5%)	36 (30.3%)	.7
Malignancy	25 (10.3%)	14 (11.4%)	11 (9.2%)	.74
Side				.72
Right	110 (45.5%)	54 (43.9%)	56 (47.1%)	
Left	127 (52.5%)	67 (54.5%)	60 (50.4%)	
Bilateral	5 (2.1%)	2 (1.6%)	3 (2.5%)	
Localization				<.001
Iliofemoral	187 (77.3%)	123 (100%)	64 (53.8%)	
Femoral	55 (22.7%)	–	55 (46.2%)	
Hemoglobin	12.19 ± 1.64	12.02 ± 1.75	12.36 ± 1.51	.11
Hematocrit	36.71 ± 4.78	36.35 ± 5.09	37.08 ± 4.43	.24
MPV	9.89 ± 1.35	9.84 ± 1.35	9.94 ± 1.36	.55
NLR	5.4 ± 3.66	5.38 ± 4.09	5.42 ± 3.16	.93
PLR	170.82 ± 59.56	170.65 ± 53.89	171.01 ± 65.14	.96

CBC = complete blood count, MPV = mean platelet volume, NLR = neutrophil lymphocyte ratio, PLR = platelet lymphocyte ratio.

**Table 2:** Comparison of the posttreatment hemoglobin, hematocrit, MPV, NLR, and PLR values between pharmacomechanical thrombectomy and medically observed patient groups.

	Total (n = 242)	Pharmacomechanic (n = 123)	Medical (n = 119)	*P* value
Posttreatment hemoglobin	12.03 ± 1.8	11.86 ± 1.8	12.22 ± 1.78	.11
Posttreatment hematocrit	36.15 ± 5.06	35.74 ± 5.22	36.58 ± 4.88	.2
Posttreatment MPV	9.77 ± 1.2	9.63 ± 1.32	9.92 ± 1.03	.06
Posttreatment NLR	4.22 ± 2.35	3.56 ± 1.93	4.9 ± 2.55	< .001
Posttreatment PLR	132.33 ± 65.45	107.59 ± 39.85	157.9 ± 76.22	< .001

MPV = mean platelet volume, NLR = neutrophil lymphocyte ratio, PLR = platelet lymphocyte ratio.

**Table 3:** Comparison of admission and follow-up MPV, NLR, and PLR values in pharmacomechanical thrombectomy and medically observed patient groups separately.

	Pharmacomechanic (n = 123)			Medical (n = 119)		
	Admission	Follow-up	*P* value	Admission	Follow-up	*P* value
MPV	9.84 ± 1.35	9.63 ± 1.32	.13	9.94 ± 1.36	9.92 ± 1.03	.84
NLR	5.38 ± 4.09	3.56 ± 1.93	< .001	5.42 ± 3.16	4.9 ± 2.55	.16
PLR	170.65 ± 53.89	107.59 ± 39.85	< .001	171.01 ± 65.14	157.9 ± 76.22	.08

MPV = mean platelet volume, NLR = neutrophil lymphocyte ratio, PLR = platelet lymphocyte ratio.

Subgroup analyses of the only iliofemoral DVT patients was performed. There was no statistically significant difference between the pharmacomechanical thrombectomy and medically observed patient groups in terms of admission MPV, NLR, and PLR values in the subgroup analyses (*P* = .774, *P* = .384, and *P* = .595, respectively). The posttreatment NLR and PLR values were significantly decreased when compared with the admission values in the pharmacomechanic thrombectomy group (*P* < .001 and *P* < .001, respectively). In subgroup analyses, there was no statistically significant difference between the admission and follow-up values of MPV, NLR, and PLR in patients treated medically (*P* = .206, *P* = .214, and *P* = .052, respectively) (Table 4).

**Table 4:** Subgroup analyses of the iliofemoral DVT patients.

	Pharmacomechanic (n = 123)			Medical (n = 64)		
	Admission	Follow-up	*P* value	Admission	Follow-up	*P* value
MPV	9.84 ± 1.35	9.63 ± 1.32	.13	9.78 ± 1.51	10.03 ± 1.15	.206
NLR	5.38 ± 4.09	3.56 ± 1.93	< .001	5.91 ± 3.48	5.23 ± 2.29	.214
PLR	170.65 ± 53.89	107.59 ± 39.85	< .001	175.18 ± 57.84	154.89 ± 76.22	.052

Comparison of the admission and follow-up MPV, NLR, and PLR values in pharmacomechanical thrombectomy and medically observed patient groups separately.

DVT = deep vein thrombosis, MPV = mean platelet volume, NLR = neutrophil lymphocyte ratio, PLR = platelet lymphocyte ratio.

## 4. Discussion

Deep vein thrombosis is an important component of VTE and one of the most common causes of mortality due to cardiovascular diseases, along with pulmonary embolism. If there is no specific treatment performed after DVT diagnosis, pulmonary embolism and post-thrombophlebitis syndrome may occur consequently.^[15]^ Although conventional anticoagulants are commonly used in the treatment of DVT, pharmacomechanical thrombectomy has been widely used as an alternative treatment. Anticoagulant therapy in DVT does not have a thrombolytic effect, and recanalization depends only on the effectiveness of the endogenous fibrinolytic system, according to Doğanci et al.^[16]^ We hypothesized that the removal of the thrombus load from the affected veins would contribute to patient recovery. Pharmacomechanical thrombectomy has several advantages over mechanical thrombectomy and other medical treatment modalities. Several studies have been conducted since catheter thrombolysis was first performed as an a thrombectomy method by Armon in 1997. The primary goal of this procedure is to reduce the thrombus load and provide venous drainage with recanalization.^[3]^ It is also as prevent pulmonary embolism and post thrombotic syndrome, which are the most undesirable complications of venous thrombus.^[17]^ The advantage of pharmacomechanical thrombectomy is that the thrombus is removed from 26% to 86% with the addition of a lytic agent.^[18]^

The pharmacomechanical thrombectomy system used in our study consisted of 4 parts: vena cava filter (Inca, Invamed, Ankara, Turkey), mechanical thrombectomy device (Mantis, Invamed, Ankara, Turkey), pharmacomechanical thrombectomy catheter (Viper, Invamed, Ankara, Turkey), and thrombus aspiration device (Dovi, Invamed, Ankara, Turkey). Inca is stationed in the IVC at the L4 level at the beginning of the procedure to prevent dislodged clots from migrating to the lungs and causing pulmonary embolism during the procedure. At the tip of the mantis catheter, a motor-operated shredder dismantles the thrombus via rotational movement and simultaneously provides pharmacological agent through the catheter. The fragmented thrombus within the lumen of the vein was removed using a Dovi aspiration system. The Viper catheter remains in the vein for at least 24 hours after the procedure is performed, preventing early blockage of the vein by administering TPA. In our opinion, the advantage of this system is that it has 4 functional catheters that differ from each other compared to other commercial market systems.

Procedure-specific complications have not been reported in the literature. Puncture, catheter, caval filter, and antithrombotic agent-related complications are the general problems associated with various types of interventional procedures. In a case report, the authors reported that the tip of the catheter, manufactured by another brand in the market, was broken and withdrawn by an embolectomy catheter.^[19]^ This type of complication occurs as a result of either foul manufacturing or improper use. Literature on pharmacomechanical thrombectomy will accumulate over time as the use of this treatment modality becomes more frequent.

The formation of clots in deep veins is the result of a series of inflammatory processes.^[20]^ Inflammatory begin within the vein after the initial wall damage of deep veins and results in thrombus formation, and consequently, inflammatory markers are increased. This biochemical state appears in laboratory tests such as CBC. Most of the tests, measuring inflammatory markers are expensive and not easily acceptable.^[21]^ The CBC test, on the other hand, is simple blood work that can be easily applied at any level laboratory and gives us precise information. Neutrophils and platelets are CBC parameters and can easily be calculated with their ratio to each other and inform us about the patient’s survey.^[11]^ We believe that patients will be more protected against unwanted complications of thromboembolism if inflammatory processes, which occur due to thrombus, are rapidly intervened and the thrombus is removed from the vein.

In a study by Kuplay et al, NLR and PLR were found to be high in iliofemoral and proximal femoral DVT. They evaluated the relation between the proximal lower extremity thrombus load and NLR and PLR values.^[22]^ In our study, similar to Kuplay et al, the NLR and PLR values of patients with iliofemoral DVT before the procedure were statistically significantly higher compared to patients with femoral DVT. The fact that the NLR and PLR values in the pharmacomechanical thrombectomy group were significantly lower than those in the medical treatment group may be due to a more effective reduction in thrombus load in the deep veins. As mentioned in the study by Akgül et al, a simple CBC test revealed precise and reliable information about thrombus load and predictions about possible clinical scenarios such as pulmonary embolism by calculating NLR and PLR values.^[23]^ Further studies will show that intravascular thrombus load and NLR – PLR values decrease faster and more effectively in pharmacotherapeutic thrombectomy compared to medical treatment, which will enlighten more clinical decision-making processes in the future.

The most important limitation of this study is its retrospective design. Another limitation was that the thrombus load in the deep venous system was not measured objectively or quantitatively at the beginning of the treatment process. Relatively small sample size is the other limitation of the study.

In conclusion, deep vein thrombosis is a disease with high mortality and morbidity rates that directly affect the quality of life of patients. We believe that fatal complications and sequelae can be prevented by timely and appropriate treatment. Pharmacomechanical thrombectomy methods are also considered to have an important advantage in reducing thrombus load more quickly and effectively. In our study, we effectively removed the thrombus load in blocked proximal veins using this method and observed a significant decrease in NLR and PLR, which are current, inexpensive, and accessible parameters.

## Author contributions

**Conceptualization:** Ali Ümit Yener.

**Data curation:** Ali Ümit Yener.

**Formal analysis:** Ali Ümit Yener, Ömer Faruk Çiçek.

**Investigation:** Ali Ümit Yener.

**Methodology:** Ali Ümit Yener.

**Project administration:** Ali Ümit Yener.

**Resources:** Ali Ümit Yener, Adnan Yalçinkaya, Özlem Yener, Ekin Can Çelik.

**Software:** Ali Ümit Yener.

**Supervision:** Ali Ümit Yener.

**Writing – original draft:** Ali Ümit Yener, Adnan Yalçinkaya, Ömer Faruk Çiçek.

**Writing – review & editing:** Ali Ümit Yener, Özlem Yener, Ekin Can Çelik, Onur Hanedan, Mustafa Cüneyt Çiçek.
